# Longitudinal changes of the femoral bone mineral density from first to third trimester of pregnancy: bone health assessment by means of non-ionizing REMS technology

**DOI:** 10.1007/s40520-023-02677-4

**Published:** 2024-02-09

**Authors:** Ruben Ramirez Zegarra, Valentina Degennaro, Maria Luisa Brandi, Greta Cagninelli, Sergio Casciaro, Gabriella Celora, Francesco Conversano, Fiorella A. Lombardi, Paola Pisani, Tullio Ghi

**Affiliations:** 1 Department of Medicine and Surgery, Obstetrics and Gynecology Unit, University of Parma, Viale A. Gramsci 14, 43126 Parma, Italy; 2 Fondazione Italiana per la Ricerca sulle Malattie dell’Osso (F.I.R.M.O.), Florence, Italy; 3 Institute of Clinical Physiology, National Research Council, Lecce, Italy; 4 Echolight Spa, Lecce, Italy

**Keywords:** Femur neck, Osteoporosis, Physiologic calcification, Bone density, Calcium metabolism, Bone development

## Abstract

**Background:**

Throughout the pregnancy, there is a substantial transfer of calcium from the maternal skeleton to the fetus, which leads to a transient net reduction of the maternal bone mineral density.

**Aims:**

To assess longitudinally the changes in the bone mineral density at the femoral neck between the first and third trimester of pregnancy in a cohort of healthy participants using Radiofrequency Echographic Multi Spectrometry (REMS) technology.

**Methods:**

Prospective, cohort study conducted at the University hospital of Parma, Italy between July 2022 and February 2023. We recruited healthy participants with an uncomplicated singleton pregnancy before 14 completed weeks of gestation. All included participants were submitted to a sonographic examination of the femoral neck to assess the bone mineral density (and the corresponding *Z*-score values) using REMS at 11–13 and 36–38 weeks of pregnancy. The primary outcome was the change in the bone mineral density values at the maternal femoral neck between the first and third trimester of pregnancy.

**Results:**

Over a period of 7 months, a total of 65 participants underwent bone mineral density measurement at the femoral neck at first and third trimester of the pregnancy using REMS. A significant reduction of the bone mineral density at the femoral neck (0.723 ± 0.069 vs 0.709 ± 0.069 g/cm^2^; *p* < 0.001) was noted with a mean bone mineral density change of − 1.9 ± 0.6% between the first and third trimester of pregnancy. At multivariable linear regression analysis, none of the demographic or clinical variables of the study population proved to be independently associated with the maternal bone mineral density changes at the femoral neck.

**Conclusions:**

Our study conducted on a cohort of healthy participants with uncomplicated pregnancy demonstrates that there is a significant reduction of bone mineral density at femoral neck from early to late gestation.

## Introduction

Pregnancy is a critical period for the maternal calcium metabolism and bone mineral status [1]. The development of the fetal skeleton requires a substantial transfer of calcium from the mother to the fetus throughout pregnancy, 80% of which is transferred during the third trimester [2]. As a result, the maternal calcium metabolism undergoes several changes to meet the high fetal demands of calcium [1, 3].

Despite the activation of several adaptive mechanisms to counterbalance calcium drainage [4–6], there is a net reduction of the bone mineral density (BMD) in both cortical and trabecular bones during pregnancy. With the use of dual-energy X-ray absorptiometry (DXA), which is the gold standard for the BMD assessment in non-pregnant populations, several studies have evaluated changes of BMD during pregnancy at axial bones, such as the femoral neck [7]. However, due to the potential harmful effects of radiation to the fetus, most of the studies assessed the maternal femoral BMD before conception and after delivery, and none of them could quantify the real reduction of BMD at the femoral neck during pregnancy [8–12]. As an alternative to DXA during pregnancy, some authors have proposed the use of quantitative ultrasonometry [13–15]. Although this technique does not emit ionizing radiation, it has been mostly used for the assessment of the bone density from peripheral skeletal sites [16]. Peripheral bones consist mainly of trabecular bone, which has a higher turnover rate than cortical bone, and hence, results of these studies cannot be generalized to bones with a higher cortical bone density, such as the femoral neck [17]. Therefore, new non-ionizing techniques are needed to evaluate the impact of the pregnancy on such bones.

Recently, the Radiofrequency Echographic Multi Spectrometry (REMS) technology has been proposed as an alternative to DXA for the assessment of the BMD at the central bone sites in non-pregnant populations. This technique has been found as reliable as DHA in the diagnosis of osteoporosis [18, 19]. Moreover, Degennaro et al. found that the assessment of the BMD at femoral neck during pregnancy by means of REMS is feasible and reported a lower BMD in pregnant compared with non-pregnant participants [20].

The aim of our study is to assess longitudinally the changes in the BMD at the femoral neck between the first and third trimester in a cohort of healthy pregnant participants using REMS technology.

## Material and methods

This was a single-center, prospective, cohort study conducted at the University Hospital of Parma, Italy, between July 2022 and February 2023. This study was performed in line with the principles of the Declaration of Helsinki. Approval was granted by the Local Ethic committee of Emilia-Romagna (Protocol #19656). This study was conducted following the STROBE guidelines [21].

Healthy participants with an uncomplicated singleton pregnancy before 14 completed weeks of gestation attending at our antenatal clinic between July and August 2022, were considered eligible for the purposes of the study. All participants reported to take regularly folic acid or multivitamins since early stages of pregnancy. Participants were approached between 11 + and 13 + 6 weeks of gestation at the time of the first trimester screening for chromosomal anomalies and enrolled if the screening yielded a low risk of major trisomies (21, 18 and 13). Written consent for study inclusion was obtained upon enrolment. Gestational age was determined at ultrasound by fetal crown–rump length measurement. Participants were not considered eligible if they were non-Caucasian or in the presence of current or previous medical conditions which could potentially interfere with the bone metabolism (e.g., thyroid, liver, kidney, bowel-disease etc.), walking disability, history of bone fractures or recent traumatic fractures, previous diagnosis of osteopenia or osteoporosis according to the Italian Society for Osteoporosis, Mineral Metabolism and Bone Diseases criteria [22], vitamin D intake > 400 IU/day, BMI > 40 kg/m^2^, age < 18 years, smoking addiction or chronic consumption of drugs including steroids or anticonvulsants.

All included participants were submitted to a sonographic examination of the femoral neck to assess the BMD by REMS technology. The sonographic assessment of the maternal femoral neck was performed by one Obstetrician with over 5 years of experience on the field (V.D.) at the end of the first trimester ultrasound screening (11–13 weeks of gestation) and repeated when the participant attended at our clinic for the standard antenatal evaluation (37–39 weeks of pregnancy). As previously described [20, 23], REMS technology consists of a simple sonographic acquisition applicable to the axial reference sites (i.e., lumbar spine and proximal femur). In the fully automatic processing of the acquired images and “raw” ultrasound signals (the so-called “radiofrequency ultrasound signals”), once the target bone interface is detected (e.g., maternal femoral neck), the corresponding bone structure and the internal region of interest is automatically analyzed. Subsequently, the algorithm integrated into the REMS device calculates the standard bone parameters which are usually provided by a DXA examination (BMD in g/cm^2^ and *Z*-score). The bone health status is assessed by comparing the obtained signal spectra with the standardized reference spectral models of osteoporotic and healthy populations [16, 24]. The femoral acquisitions were performed using an EchoStation device (Echolight Spa, Lecce, Italy) equipped with a convex probe operating with a frequency of 3.5 MHz according to the standard procedure. More specifically, a 40-s software-guided ultrasound scan was performed with the ultrasound probe placed on the head–neck axis of the maternal femur and then parallel to the femur long axis [18]. To ensure diagnostic reliability, all clinical data collected during the maternal REMS acquisitions underwent a quality control by two experienced operators to identify possible errors. REMS errors were identified as deviations from the acquisition procedure described in the EchoStation user manual: they were typically associated with wrong or suboptimal settings of transducer focus and/or scan depth, or with incomplete adherence to the on-screen and audible indications provided by the software. If the image did not meet all the requirements, the participant was excluded. Moreover, participants were also excluded from the study group if any of the following conditions occurred between the two ultrasound examinations: abortion, spontaneous or indicated preterm birth, intrauterine fetal death, postnatal diagnosis of congenital anomalies, pregnancy complications (i.e., hypertensive disorders, gestational diabetes, cholestasis, gestational hypothyroidism), the need of medications that may interfere with bone metabolism such as vitamin D intake > 400 IU/day, heparin or corticosteroids, delivery in a different hospital.

Clinical data were retrieved from the medical records of each participant. Maternal data included age, ethnicity, body mass index (BMI) at booking, maternal height, parity, smoking status and comorbidities. Data were recorded and stored in a Microsoft Excel (Microsoft, Redmond, WA, USA) secured pseudonymized database, which was accessible only by the members of the research team.

The main outcome of the study was the change in the BMD of the maternal proximal femur between the first and third trimester of pregnancy.

### Statistical analysis

In this study, the sample size was calculated based on the number of childbirths/year at the University Hospital of Parma (which is equal to a population of about 2600 childbirths/year). We aimed at determining a sample size that was representative of the whole population of pregnant people typically delivering at this hospital in 1 year with a confidence level (expressed as a percentage) of 95% and a confidence interval (also called margin of error) of 15%. By employing the sample size calculator available at https://www.surveysystem.com/sscalc.htm, our sought sample size resulted equal to 42, which was then multiplied by a safety factor of 1.5 and resulted in the final value of 63.

Statistical analysis was performed using Statistical Package for Social Sciences (SPSS) v. 22 (IBM Inc., Armonk, NY, USA). The Kolmogorov–Smirnov test was used to assess the normality of the distribution of the data. Statistical analysis was performed using the Chi-square test for categorical variables and the Student’s *t* test for continuous variables. The results were presented as number (percentage) or mean ± standard deviation (SD). Multivariable linear regression analysis was used to control for potential confounding variables. *P* < 0.05 was considered as statistically significant.

## Results

**Fig. 1:**
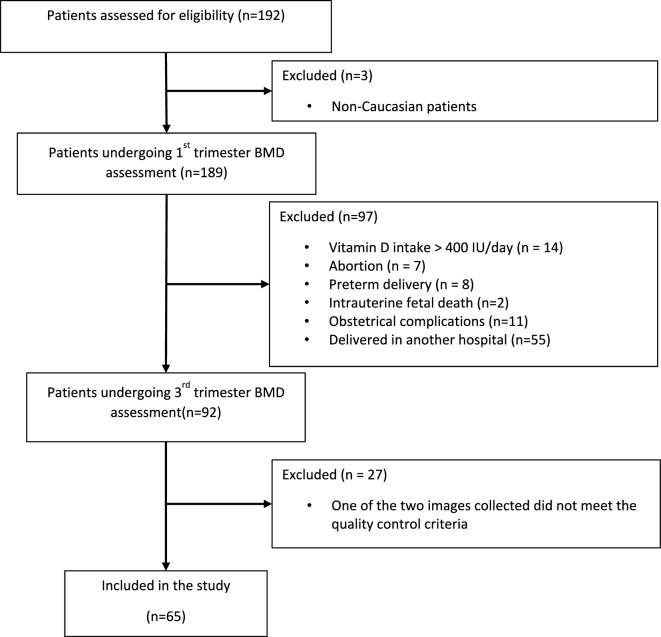
Flowchart (according to the STROBE guideline) of patient enrollment of a cohort of health pregnant women for the assessment of the maternal bone mineral density (BMD) at femoral neck at first and third trimester of pregnancy

Over a period of 7 months, a total of 189 participants were found eligible for the study purposes, were enrolled and underwent BMD measurement at the femoral neck in the first trimester. During the period between the first and third trimester examination, 14 participants started on vitamin D intake > 400 IU/day, seven had an abortion, eight preterm delivery, two intrauterine fetal death, 11 obstetrical complications and 55 decided to deliver in a different hospital: and were consequently excluded, leaving 92 for the assessment of the BMD at femoral neck in the third trimester. Lastly, 27 participants were excluded from the analysis because one out of the two REMS scans did not meet the quality standards, leaving a total of 65 low risk participants with uncomplicated pregnancy who completed the two steps bone assessment (1st and 3rd trimester) for the final data analysis (Fig. 1). The main demographic and clinical features of the study group are shown on Table 1. All participants were Caucasian, with a mean age of 33.7 ± 4.6 years, a mean BMI at booking of 22.1 ± 3.1 kg/m^2^, and a BMI at third trimester of 25.8 ± 2.9 kg/m^2^.

**Table 1:** Demographic characteristics of the 65 patients included in our study that underwent assessment of the maternal bone mineral density (BMD) at femoral neck at first and third trimester of pregnancy

Variable	All cases *N* = 65
Maternal age, years mean ± SD	33.7 ± 4.6
Ethnicity, *N* (%) Caucasian	65 (100.0)
Maternal height mean ± SD	164.7 ± 6.1
Maternal weight at booking mean ± SD	59.9 ± 8.8
Maternal weight at 3rd trimester mean ± SD	70.2 ± 9.2
Maternal weight change mean ± SD	10.3 ± 4.3
Maternal BMI at booking, kg/m^2^ mean ± SD	22.1 ± 3.1
Maternal BMI at 3rd trimester, kg/m^2^ mean ± SD	25.8 ± 2.9
Maternal BMI change mean ± SD	3.7 ± 1.5
Nullipara *N* (%)	44 (67.7)

Data are given as mean ± standard deviation (SD) or number (percentage)

*BMI* body mass index

**Fig. 2:**
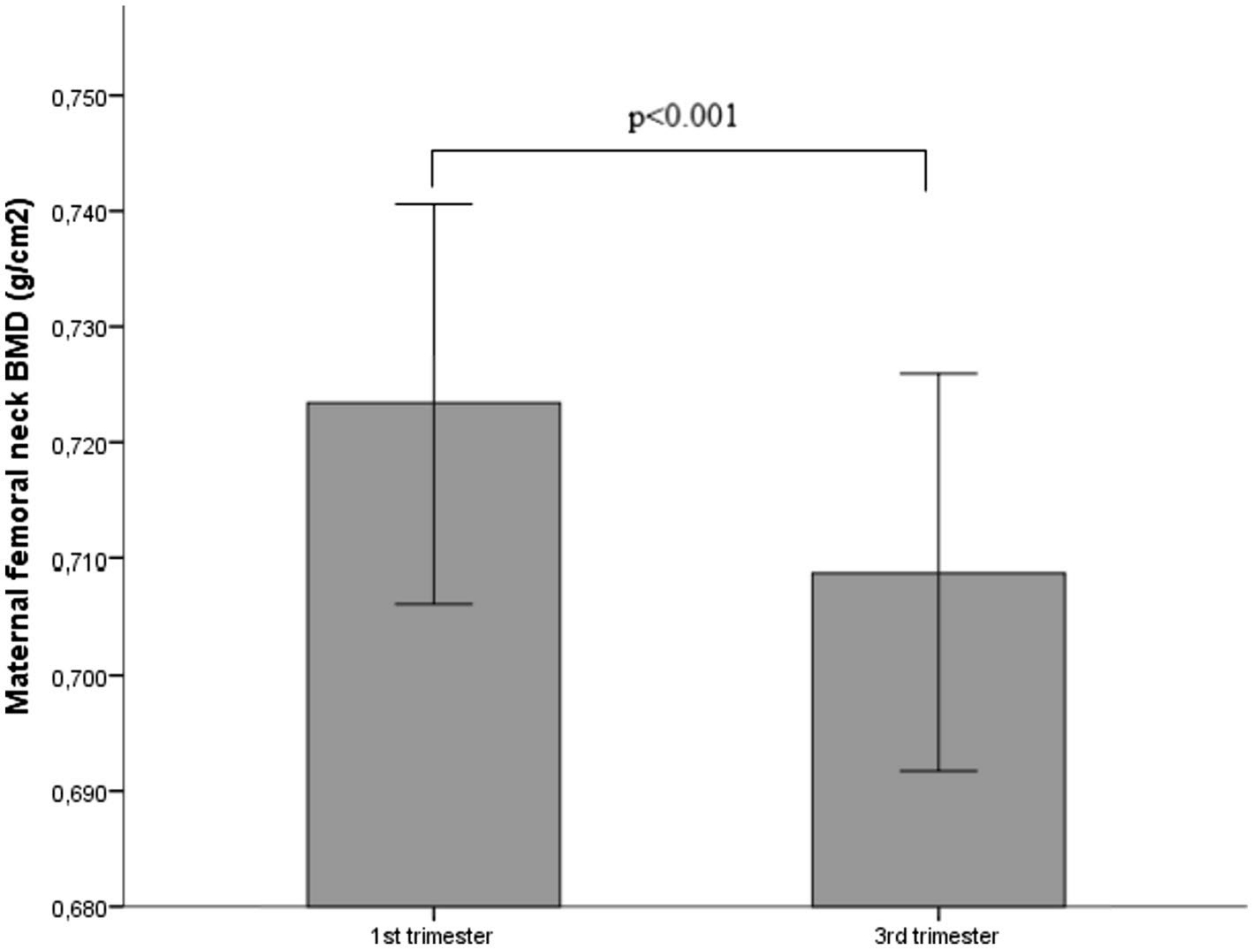
Bar graph showing the mean bone mineral density (BMD) at maternal femur neck using REMS in a cohort of 65 pregnant women at first and third trimester

**Fig. 3:**
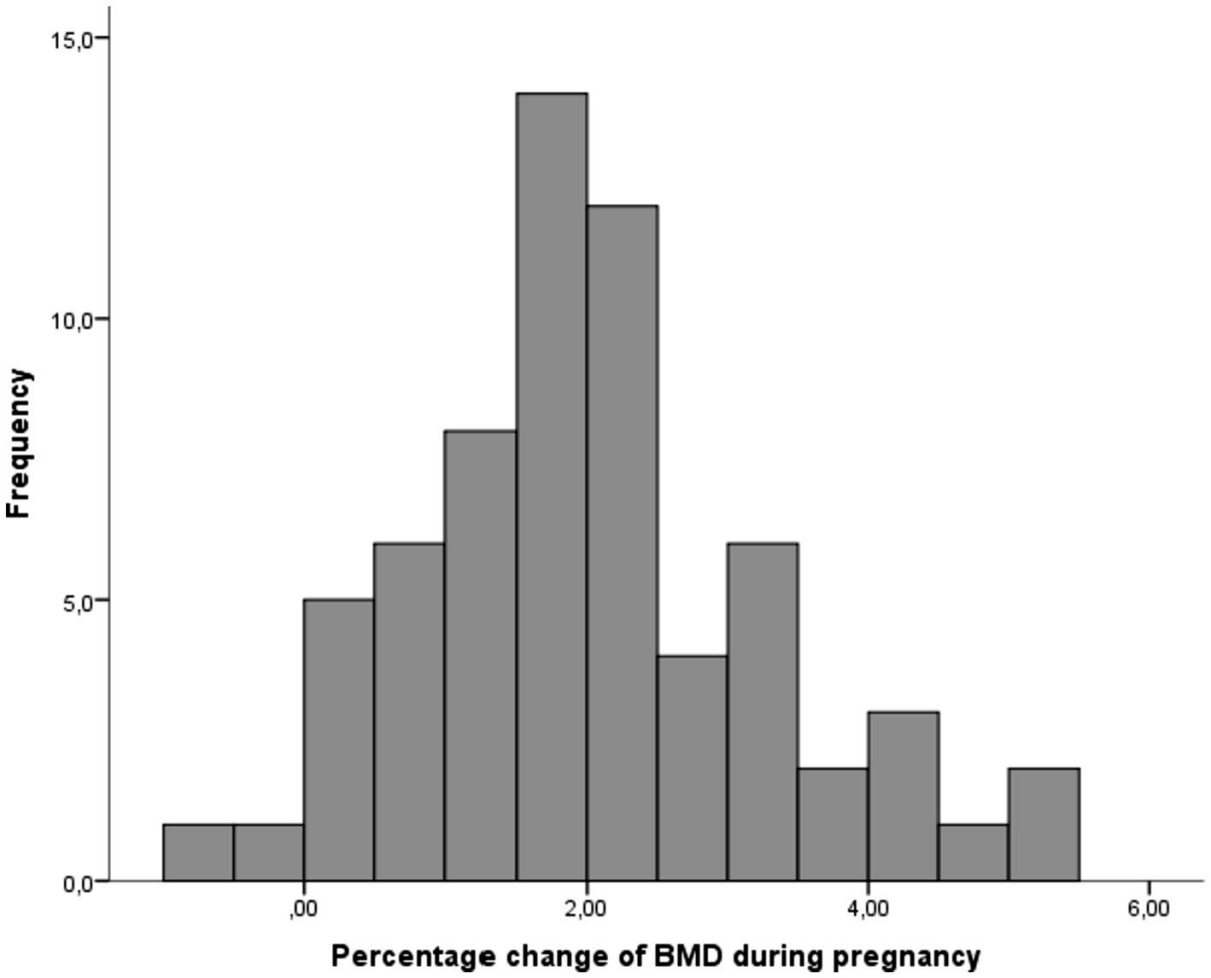
Histogram of the percentages of change of maternal bone mineral density (BMD) at femoral neck assessed using REMS technology between the first and third trimester of pregnancy

From the first to the third trimester of pregnancy, there was a significant reduction in BMD at the femoral neck (0.723 ± 0.069 vs 0.709 ± 0.069 g/cm^2^; *P* < 0.001) with a mean change of − 1.9 ± 0.6% (Table 2 and Fig. 2). More in detail, 63 participants of our cohort experienced a reduction of the BMD throughout the pregnancy, with a maximal reduction of 5.5%. In contrast, two participants from our cohort showed a slight increase of 0.2% and 0.8% in the BMD at femoral neck between the first and third trimester of pregnancy (Fig. 3).

**Table 2:** Maternal bone mineral density (BMD) and *Z*-scores at femoral neck at first and third trimester of pregnancy

	First trimester	Third trimester	Percentage ∆	*P*
BMD (g/cm^2^)	0.723 ± 0.069	0.709 ± 0.069	− 1.9 ± 0.6%	< 0.001
*Z*-score	− 1.0 ± 0.6	− 1.1 ± 0.6		< 0.001

Data are given as mean ± standard deviation

At multivariable linear regression analysis, BMI, parity, maternal age and maternal height had no significant effect on the changes in the maternal BMD at femoral neck (Table 3).

**Table 3:** Multivariable linear regression analysis assessing the effect of demographic variables on changes in the maternal bone mineral density at femoral neck between the first and third trimester of pregnancy

Variable	*B*	SE	Beta	*P*	95% CI for *B*
Constant	0.0023	0.0359		0.95	− 0.070 to 0.074
Maternal age	0.0003	0.0003	0.15	0.27	− 0.001 to 0.001
Parity	0.0010	0.0027	0.05	0.71	− 0.004 to 0.006
Maternal height	0.0001	0.0001	0.03	0.84	− 0.001 to 0.001
Body mass index	− 0.0014	0.0008	− 0.22	0.11	− 0.003 to 0.001

*CI* confidence intervals

## Discussion

To our knowledge, the present study is the first to determine and quantify longitudinally the changes of the maternal BMD at the femoral neck in a cohort of healthy pregnant participants. In this pilot study, we have demonstrated that there is a net reduction of the maternal BMD between the first and third trimester of pregnancy, as testified by a 2% decrease of the maternal BMD at the femoral neck detected using REMS technology.

Throughout the pregnancy and especially during the third trimester, there is a substantial transfer of calcium (around 30 g of calcium) from the maternal skeleton to the fetus to assure fetal bone growth and mineralization [2]. In response, several maternal adaptive mechanisms are activated to attenuate maternal calcium drainage [1, 3]. However, the increased calcium resorption during the third trimester seems to overcome the compensatory mechanisms and leads to a transient net reduction of the BMD at the femoral neck, as demonstrated by our results. Little is known about the actual BMD changes in pregnancy at axial bones, such as the femoral neck. The femoral neck is the only central site consisting of a high cortical bone density that is accessible during pregnancy, as the assessment of maternal spine bone mineral density using REMS might not be possible due to the presence of the gravid uterus. Due to the potential harms of ionizing radiation to the fetus, most of the studies using DXA (i.e., the gold standard) have assessed the maternal femoral BMD at preconception and after delivery. These studies have reported a 3–4% decrease in maternal femoral BMD after delivery compared to the values obtained before conception [8–12], which are similar to our results. So far, only one study evaluated the maternal femoral BMD during pregnancy using a new low-radiation DXA: Wei et al. assessed longitudinally the BMD at the femoral neck in participants between 12 and 20 weeks of gestation (first measurement) and the postpartum period (second measurement), reporting a change in the BMD at femoral neck of − 0.01 g/cm^2^ [25], which is consistent with our results.

Studies using quantitative ultrasonometry, as a safer alternative to DXA, have also reported a net reduction of the maternal bone density at the peripheral bones, especially, at the calcaneus [13–15]. However, the main limitation of this technique is that it can only be used to assess peripheral skeletal sites and is not very representative of the changes occurring at the axial bones [16]. Trabecular bone, the main component of peripheral bones, has a higher turnover rate than cortical bone [17]. This might result in lower BMD values by the end of the pregnancy compared to axial bones, such as the femoral neck, which has a higher cortical bone density. This difference can be more clearly depicted when comparing our results with the study conducted by To et al. [13], in which the authors reported a BMD decrease of 6% at the calcaneus between the first and third trimester of pregnancy, a value which is threefold higher than the one we reported at the femoral neck. Moreover, the World Health Organization defined the skeletal axial sites (femoral neck and lumbar spine) as the reference anatomical sites to assess the overall level of BMD in a subject [26].

The impact of maternal demographics, especially BMI and parity is still a matter of debate. While some studies have found that BMI [25] and parity [27] are associated with BMD changes, others could not confirm these findings [13, 15]. Consistent with the latter studies, we did not find any association between the maternal demographics and the BMD changes during pregnancy even after adjusting for confounders.

REMS has emerged as an alternative to DXA for the assessment of axial skeletal sites, because it is low cost, radiation free and easily accessible. Moreover, it has a similar accuracy for the BMD assessment in non-pregnant women compared to DXA [18, 19]. Recently, Degennaro et al. demonstrated that the use of REMS for the assessment of the BMD at the femoral neck during pregnancy is feasible, reporting a lower maternal BMD in pregnant compared to non-pregnant participants [20]. The introduction of the REMS technology for the assessment of the BMD during pregnancy may represent a safe opportunity for monitoring the bone health of patients during pregnancy, with the peculiar advantage of allowing the measurements at axial skeletal sites. Although the reduction of maternal bone mass during pregnancy seems to be transient, it increases the risk of osteopenia and bone fragility, and in few cases, it might even progress to osteoporosis [28, 29]. These patients might, therefore, benefit from serial measurements of the BMD, which would be preferable to be performed by employing non-ionizing imaging techniques such as REMS. However, further studies are needed to evaluate the impact of these serial measurements on the prevention of osteopenia. REMS might also be used to understand the impact of some medicaments used during pregnancy on the maternal BMD. Heparin, glucocorticoids and anti-epileptic drugs are associated with bone loss in non-pregnant populations [30]. Nevertheless, due to the lack of dedicated and harmless techniques, it has not been possible so far to quantify the associated BMD changes during pregnancy. REMS would allow a more tailored follow-up of these patients and a better management of the bone loss associated with these medicaments.

Evidence on the impact of vitamin D [25, 31] and calcium supplementation [32, 33] on the reduction of maternal bone mass is still contradictory. In such context, REMS represents the first opportunity to study the exact role of calcium and vitamin D supplementation on the maternal skeleton, as the BMD could be longitudinally assessed throughout pregnancy in patients taking these supplements. Moreover, this might allow obstetricians to better tailor the administration of vitamin D and calcium based on a real quantification of the maternal BMD.

The main strength of our study is represented by its original and prospective design, including strict exclusion criteria. Thus, our results might offer a good insight on the real changes of maternal BMD during pregnancy in healthy participants. We also acknowledge some limitations to our study. Our results might not extrapolate to non-white populations, as all our participants were of white ethnicity. African people tend to have higher BMD values, whereas Asian people lower BMD values compared to Caucasian ones [34, 35]. Another limitation is the relatively small number of participants recruited for study purposes. This is due to the low number of healthy low-risk women attending our tertiary care center during the first trimester. The exclusion of 27 out of 92 participants could be seen as a limitation. However, participants were excluded because one of the two obtained images (from the first or third trimester) did not meet the quality criteria. If we convert this numbers to images, we had in total 184 images coming from 92 participants, of which 27 (14.7%) were excluded. This is consistent with previous studies and should not be seen as a limitation [18, 36]. Finally, some variables that might impact the BMD of pregnant populations, such as diet, physical activity or the exact quantity of calcium intake, were not assessed and should be addressed in further studies.

Our study, conducted on a cohort of healthy participants with uncomplicated pregnancy, demonstrates that there is a significant reduction of the BMD at the femoral neck from early to late gestation. Based on this preliminary evidence, REMS might become an important tool in the assessment and monitoring of the BMD during pregnancy. Furthermore, our study opens new perspectives for understanding the impact of certain therapies on the maternal bone mass.

## Data Availability

The data that support the findings of this study are not openly available due to reasons of sensitivity and are available from the corresponding author upon reasonable request. Data are located in controlled access data storage at the University of Parma, Italy.
